# Prognostic Role of Receptor Tyrosine Kinase–Like Orphan Receptors in Intestinal-Type Gastric Cancer

**DOI:** 10.31557/APJCP.2021.22.7.2125

**Published:** 2021-07

**Authors:** Rajeev Nema, Priti Patel, Ashok Kumar

**Affiliations:** *Department of Biochemistry, All India Institute of Medical Sciences (AIIMS) Bhopal, Saket Nagar, Bhopal, India. *

**Keywords:** ROR1, ROR2, WNT5A, NKX2-1, FOXF1, prognosis, KM Plotter

## Abstract

**Background:**

Gastric cancer (GC) is diagnosed at advanced stages and has high mortality rates. Surgical resection and adjuvant chemotherapy are the main therapeutic approaches for GC. Despite curative resection, recurrence and metastasis contribute to a high mortality rate in patients with GC. The receptor-tyrosine-kinase-like orphan receptors 1/2 (*ROR1/2*) are transmembrane proteins belonging to the receptor tyrosine kinase (RTK) family. ROR1 and ROR2 are known to overexpress in the tumor tissues from several types of cancer patients. However, the role of RORs in the prognosis has not been understood.

**Methods:**

This study aimed to determine the association of mRNA expression of ROR1, ROR2, and their signaling components WNT5A, NKX2-1, and FOXF1, with the survival outcome of GC patients. We performed Kaplan-Meir survival analysis on publicly available ‘The Cancer Genome Atlas (TCGA)’ data sets using ‘Kaplan-Meir Plotter.’

**Results:**

High mRNA expression of *ROR1, ROR2, NKX2-1*, and *FOXF1* was significantly correlated with worse overall survival (OS) of GC patients. Interestingly ROR1 and ROR showed a prognostic role in the intestinal subtype, but not in the diffuse subtype of GC. Furthermore, ROR1 was positively correlated with regulatory T cells and M2-type macrophages and negatively correlated with Th17 and natural killer T cells in the tumor stroma of patients with GC.

**Conclusion:**

We conclude that the expression of ROR1, ROR2, and their associated genes correlate with worst prognosis of GC patients, particularly in the intestinal type.

## Introduction

Gastric cancer (GC) remains a significant global public health problem, and it has a poor 5-year survival rate of less than 30% (Rawla and Barsouk, 2018). According to the Globocan data, in 2018, GC caused the deaths of 780,000 people (Bray et al., 2018). According to Lauren’s classification, GC is categorized into intestinal and diffuse types; both the types differ in responsiveness towards drugs, recurrence, and prognosis (Hu et al., 2012). Molecular pathway networks are altered in intestinal- and diffuse-type GC, and each subtype has a unique gene expression signature (Tanabe et al., 2020). Despite curative resection, recurrence and metastasis contribute to a high mortality rate in GC patients (Rawla and Barsouk, 2018). Unfortunately, immune-therapies based on chimeric antigen receptor T cells (CAR-T cells), dendritic cells (DCs), and immune checkpoints are not always effective due to tumor heterogeneity and the complicated tumor microenvironment (TME). Identifying novel therapeutic targets and prognostic indicators are therefore highly desirable because potential therapeutic targets remain limited.

The receptor tyrosine kinases (RTK)-like orphan receptors (RORs), are members of the RTK family (Borcherding et al., 2014). Both the RORs regulate various processes, including cell proliferation, survival, and metastasis (Du and Lovly, 2018). ROR1 is overexpressed in hematological malignancies and solid cancers, including colon, lung, and pancreatic cancers (Diamanti et al., 2019; Enayati et al., 2019). ROR2 overexpresses in several types of solid malignancies, including osteosarcoma, renal, and breast cancer (G et al., 2012; Dai et al., 2017; Guo et al., 2020a). ROR2 mediates epithelial-to-mesenchymal transition (EMT) in breast cancer through p38/mitogen-activated protein kinase (Xu et al., 2020). Hence, RORs are an attractive and promising therapeutic target for the management of cancers.

Mechanistically, both *ROR1* and *ROR2* act as a receptor or a co-receptor for Wnt5a that can bind extracellular cysteine-rich domain, initiating intracellular signaling cascades (Menck et al., 2021). Although Wnt5a signaling regulates vital developmental processes, including proliferation, differentiation, migration, adhesion, and polarity (Menck et al., 2021). However, the aberrant activation or inhibition of Wnt5a signaling is emerging as an important event in cancer progression, exerting both oncogenic and tumor-suppressive effects (Astudillo, 2020; Menck et al., 2021). Recent studies have shown a correlation between ROR1 expression and poor clinical outcomes, including relapse and survival in ovarian cancer patients (Zhang et al., 2017). Furthermore, Wnt5a induces ROR1 and ROR2 expression in esophageal squamous cell carcinoma to activate RhoA (Wu et al., 2019). In addition, Hedgehog signaling has been shown to induce the transcriptional activation of Wnt5A via forkhead box transcription factor F1 (FOXF1) (Katoh and Katoh, 2009). It induces EMT and metastasis in several cancer types, including GC (Ran et al., 2018; Gu and Hu, 2019). ROR1 is a transcriptional target of NKX2-1, which maintains a sustained balance between pro-survival and pro-apoptotic signaling by inducing ROR1 expression (Yamaguchi et al., 2012; Borcherding et al., 2014). However, the role of ROR1, ROR2, and their associated molecules Wnt5A, NKX2-1, and FOXF1 have not fully been explored in GC. 

In the present study, we have analyzed the prognostic role of genes involved in Wnt5A-ROR signaling in GC using web-based bioinformatics on publicly available ‘The Cancer Genome Atlas (TCGA) datasets. Further, we have analyzed the correlation of ROR signaling genes with the survival outcome of the GC patients using the Kaplan-Meier (KM) plotter. We found that the expression of *ROR1, ROR2, NKX2-1*, and *FOXF1 *was significantly correlated with worse OS of patients with GC. ROR1 and ROR2 showed high prognostic value in the intestinal types but not in the diffuse types. Analysis of tumor-infiltrating immune cells (TIICs) from GC showed that ROR1 was significantly associated with regulatory T cells (Tregs), natural killer T cells (NKT), Th17 cells, and M2-type macrophages in the tumor stroma of patients with GC. Thus,* ROR1, ROR2, NKX2-1*, and *FOXF1 *could be further validated as prognostic markers of GC.

## Materials and Methods

*Survival Outcome Analysis*

The KM plotter (kmplot.com) is a widely used database for the meta-analysis of publicly available TCGA microarray datasets to identify prognostic biomarkers (Győrffy et al., 2013). KM plotter contains the GC datasets from TCGA, Cancer Biomedical Informatics Grid (caBIG), and the Gene Expression Omnibus (GEO). The KM plotter has been used to identify the genes that could serve as potential prognostic markers for relapse-free survival (RFS), distant metastasis-free survival (DMFS), OS, and post-progression survival (PPS) in a variety of cancers (Wu et al., 2016; Wang et al., 2019; Han et al., 2020). We used the KM plotter to determine the association between gene-specific mRNA expression and OS of GC patients (Győrffy et al., 2013). In KM plotter, gene expression data and clinicopathological features such as tumor-node-metastasis (TNM) staging, Lauren classification, and HER2 status are integrated simultaneously by a Postgres SQL server (Szász et al., 2016). Currently, in KM plotter, microarray gene expression data from 1440 GC patients is available. Further, the KM plotter database has survival outcome data for 881 (OS), 645 (FP), and 503 (PPS) GC patients for a follow-up period of 12.5 years.

Gene names including *ROR1, ROR2, WNT5A, NKX2-1*, and* FOXF1 *were entered into the database to obtain KM survival plots as described previously (Nema et al., 2021). Affymetrix IDs of all the genes analyzed in this study are shown in Supplementary Table 1. A median survival checkbox was selected for mRNA expression to divide patients into high expression and low expression groups. Hazard ratio (HR) with 95% confidence intervals (CI) and log-rank P were calculated by the KM plotter. P<0.05 was considered statistically significant. To analyze the combined effect of multiple genes on the survival outcome of patients with GC, we performed multivariate analysis.

In addition, the KM plotter also has RNA-Sequencing data from 7462 patients in 21 different cancer types acquired from the TCGA repository. Correlations between gene expression and survival were computed using the Cox proportional hazards regression and by plotting Kaplan-Meier survival plots (Nagy et al., 2020). 

*Association of ROR1 with TIICs*

EMTome is a web-based computational tool for the evaluation of pan-cancer analysis of EMT genes and signatures (Vasaikar et al., 2021). EMTome database contains multi-omics immune data from TCGA and provides a comprehensive analysis of infiltrating immune cells. Association between the ROR1 with TIICs (CD4+, CD8+ T cells, Th17, NKT, macrophages, and Treg cells) were selected for correlation by EMTome. Further, Tumor Immune Estimation Resource (TIMER) 2.0 (timer.cistrome.org), a bioinformatics tool, was used to determine the association between the expression of the ROR1 gene with TIICs (Li et al., 2017). Tumor purity was taken into consideration while calculating Spearman’s correlation. 

*Genomic Alteration Analysis*

The cBioPortal (http://cbioportal.org/ ) is a publicly accessible cancer genomics portal to explore, visualize, and analyze multidimensional cancer genomics data (Gao et al., 2013). The portal contains many published cancer studies, including CCLE and TCGA. It contains genomic sequencing datasets from 1512 GC patients from seven published studies (Gao et al., 2013). Genomic alterations summary ‘OncoPrint’ was used to represent the genomic alterations of Wnt5A-ROR1/2 signaling genes, including *ROR1, ROR2, WNT5A, NKX2-1*, and *FOXF1 *in stomach adenocarcinoma (STAD) datasets. 

## Results

**Figure 1:**
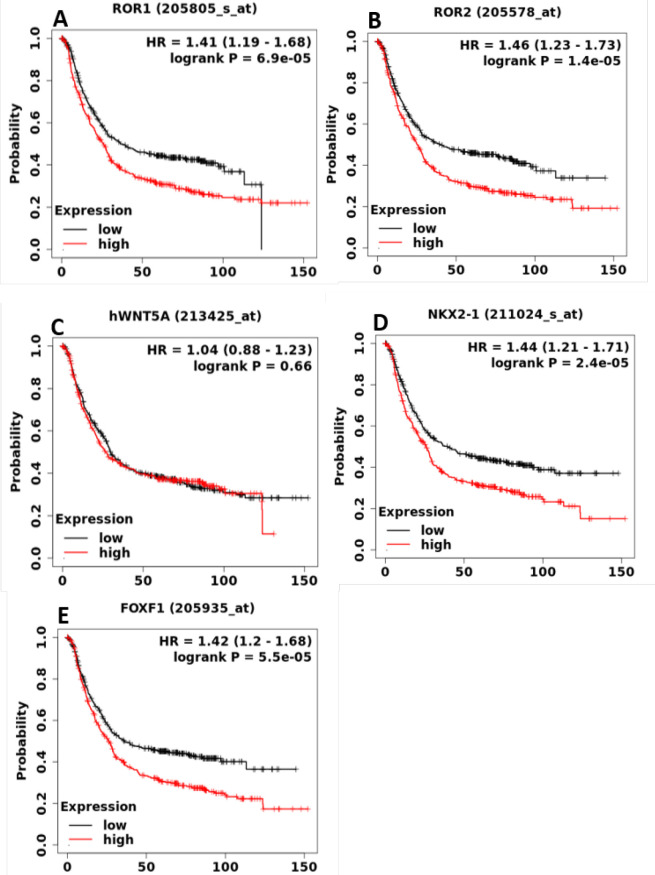
Prognostic Role of mRNA Expression of Wnt5a-ROR Signaling Genes in GC Patients. The Kaplan-Meier (KM) survival curves (overall survival) were plotted for *ROR1, ROR2 WNT5A, NKX2-1* and *FOXF1* genes from publicly available KM plotter database (N= 875 GC patients)

KM survival curves were plotted for all the GC patients (N = 1440) with the genes mentioned above. As shown in Figure 1, except WNT5A, mRNA expression of all the genes was significantly associated with worse OS in GC patients. Among all the genes analyzed in this study, ROR2 showed the best prognostic value (HR = 1.46, 95% CI: 1.23–1.73) (Figure 1B). To ascertain the role of Wnt5a-ROR signaling genes with other survival outcomes, we also drafted KM plots for FP and PPS (Table 1). Among all the genes analyzed, ROR1 exhibited a highly significant (HR = 2.15, 95% CI: 1.71–2.70) association with FP and PPS in patients with GC (Table 1). An approximately two-fold difference in the median PPS was noted between the patients with high expression of ROR1 and low expression of ROR1. Then, the KM curves were plotted for male and female GC patients separately for each gene. All the four genes showed close association with the OS with male and female GC patients (Supplementary Figure S1).

**Figure 2:**
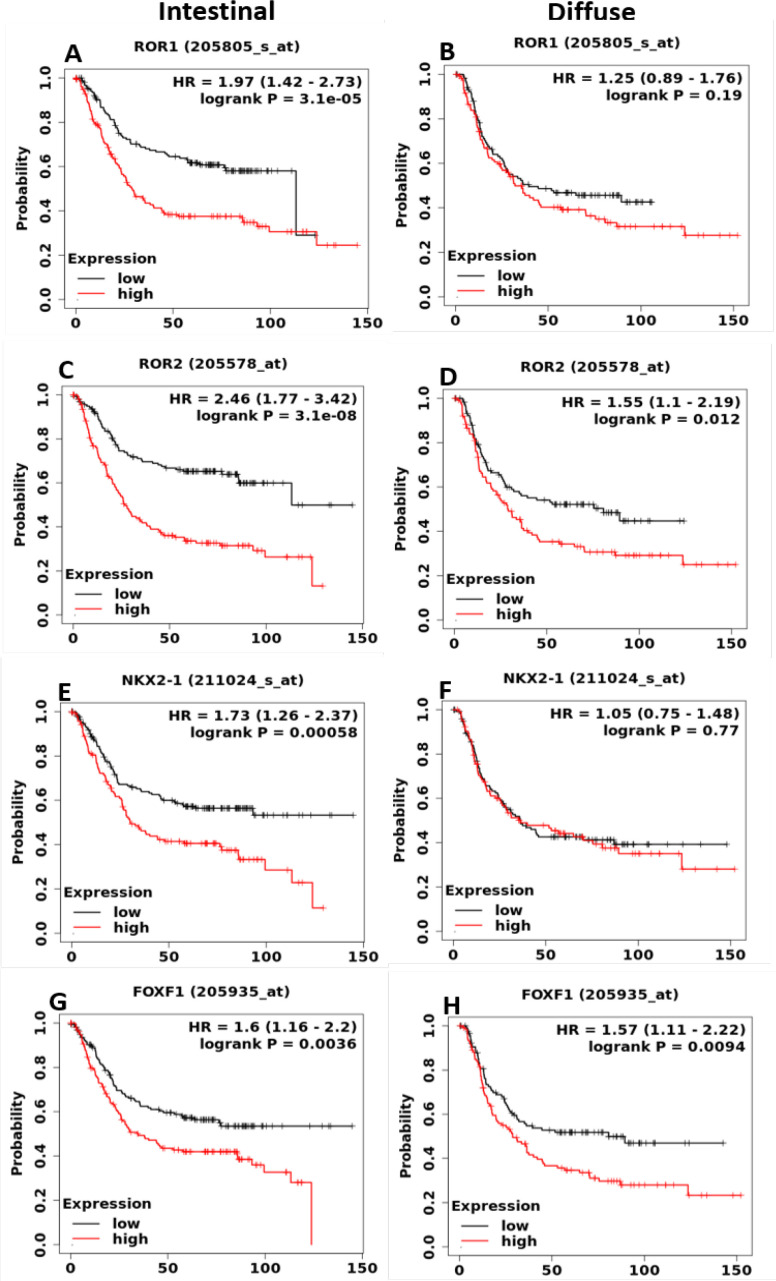
Prognostic Role of mRNA Expression of Wnt5a-ROR Signaling Genes in Histological Subtypes of GC patients. The KM survival curve were plotted for *ROR1, ROR2, NKX2-1* and *FOXF1* genes for intestinal and diffuse types of GC (Intestinal N= 336 and Diffuse N=248)

Then, we explored the prognostic role of Wnt5a-ROR signaling genes in the different histological subtypes of GC. As shown in Figure 2A-B, ROR1 exhibited high prognostic value in the intestinal subtype but not in the diffuse subtype of GC. Similarly, ROR2 showed a highly significant prognostic value in the intestinal subtype (HR = 2.46, 95% CI: 1.77–3.42), compared to diffuse subtype (HR = 1.55, 95% CI: 1.1–2.19) (Figure 2C-D). Notably, in the intestinal type of GC, the approximately four-fold difference in the median OS was noted between the patients with high expression of ROR2 versus low expression of ROR2 (high expression cohort, median OS = 26.8 months versus low expression cohort, median OS = 113.2 months). A subtype-specific prognostic value of NKX2-1 was observed in the patients with GC; the former showed a high prognostic value in the intestinal subtype but not in the diffused subtype (Figure 2E-F). FOXF1 showed a comparable prognostic value in the OS of both the subtypes of GC patients (Figure 2G-H). Importantly, ROR2 also exhibited prognostic value in the advanced stages of GC (Supplementary Figure S2).

**Figure 3:**
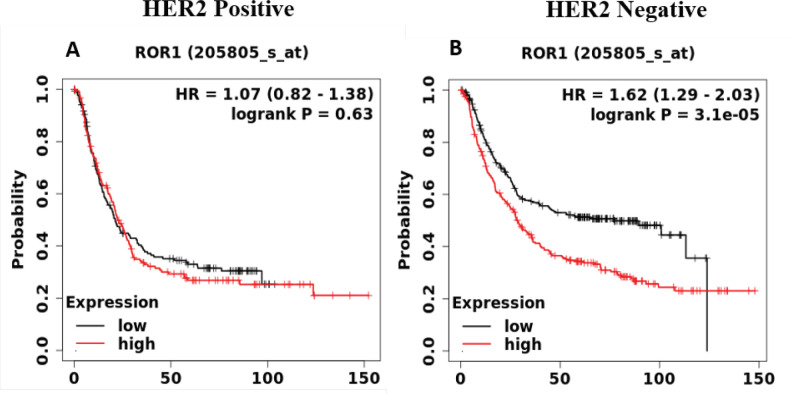
The KM Survival Curves were Plotted for *ROR1 *Gene in HER2-positive and HER2-negative GC patients (HER2 Negative N= 532 and HER2 Positive N= 343 GC patients)

Approximately 15% to 20% of advanced GC patients show HER2 positivity due to its overexpression or amplification. Intestinal-type GC show HER2 positivity more commonly compared with diffuse-type or mixed-type cancers (Joshi and Badgwell, 2021). We compared the prognostic role of Wnt5a-ROR signing genes in HER2-positive and HER2-negative GC patients. As shown in Figure 3A-B, there was a differential association of ROR1 expression with the survival outcome of GC patients with or without HER2 expression. HER2-negative GC patients with lower expression of ROR1 had a much better chance of survival than the GC patients with high expression of ROR1. However, ROR1 expression did not show any apparent difference in the median OS in the GC patients with HER2-positive status. Except for WNT5A, other genes of the Wnt5a-ROR pathway showed significant association in GC patients irrespective of HER2 status (Supplementary Figure S3 A-H).

**Figure 4:**
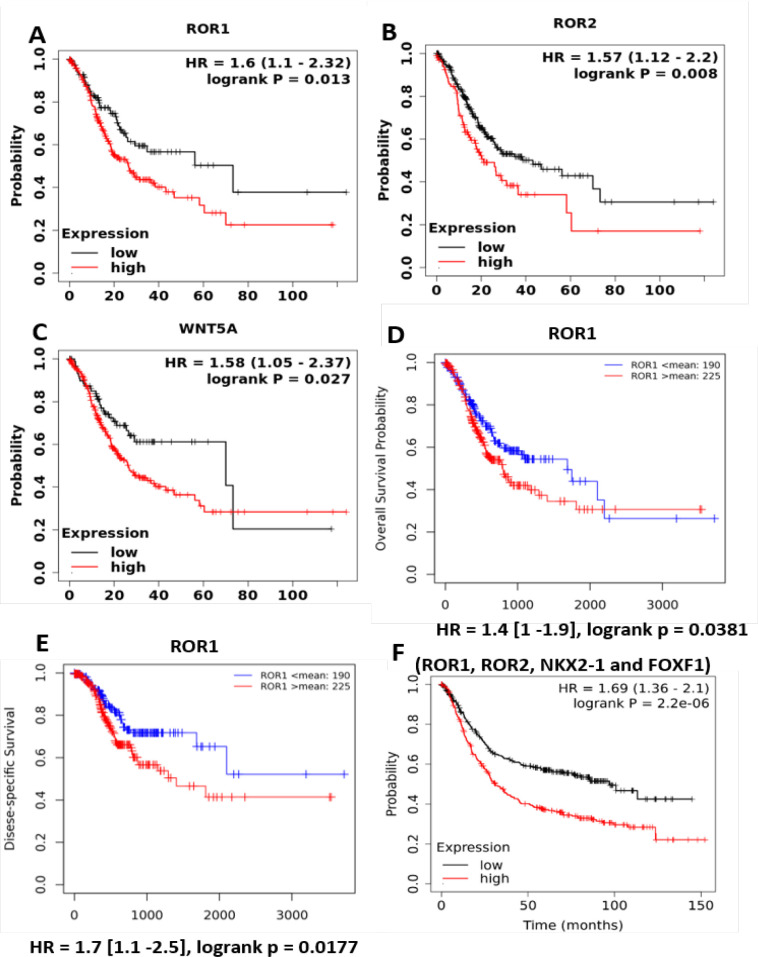
Prognostic Role of mRNA Expression (RNA-seq) of *ROR1, ROR2* and *WNT5A* Genes (N=375). The KM survival curves were plotted for *ROR1, ROR2* and *WNT5*A using pan-cancer RNA-seq data with GC patients (N=375)

Furthermore, to validate the prognostic role of the Wnt5a-ROR signaling pathway in GC, a pan-cancer RNA-Seq option from the KM plotter was used (Nagy et al., 2020). As shown in Supplementary Table 2, ROR1 signaling genes showed a significant prognostic role in several cancers, including GC. RNA-Seq data analysis corroborated well with the gene chip data (microarray). Genes of Wnt5a-ROR signaling pathway, except NKX2-1 showed a significant association with OS in the patients with GC (ROR1, HR = 1.6, 95% CI: 1.1–2.32; ROR2, HR = 1.57, 95% CI: 1.12–2.2; WNT5A, HR = 1.58, 95% CI: 1.05–2.37) (Figure 4 A-C). A similar association of ROR1 with OS and DFS was obtained with the EMTome database (Figure 4 D-E).

A multiple gene option was chosen in the KM plotter to determine the combined effect of the Wnt5a-ROR signaling genes. As shown in Figure 4F, ROR1, ROR2, NKX2-1 and FOFX1 together showed a high prognostic value in GC (HR = 1.69, 95% CI: 1.36–2.1). More than a three-fold difference in the median OS was noted between the patients with high expression of above genes versus low expression of above genes (high expression cohort, median OS = 31.1 months versus low expression cohort, median OS = 97 months).

*ROR1 expression correlates with TIICs*

**Figure 5:**
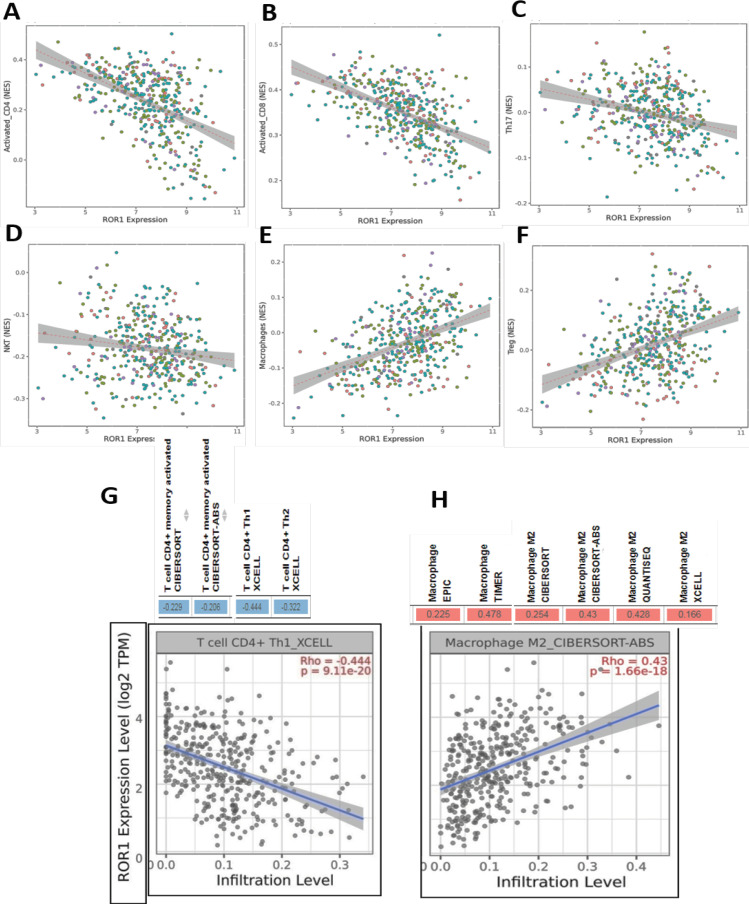
Association of ROR1 with TIICs was Determined by EMTome in GC Patients (A-F) A–D, ROR1 was Negatively Correlated with Activated CD4+, Activated CD8+, Th17 and NKT E–F, ROR1 was Positively Correlated with Treg and Macrophages, G-H, TIMER 2.0 was used to Determine the Association of ROR1 with Tregs and Macrophage Subtypes

The infiltration of immune cells in the TME shows a positive correlation with the survival of patients with malignancies (Wang et al., 2011; Liu et al., 2015). Different types of TIICs can predict prognosis more accurately than the TNM stages. Indeed, higher intra-tumoral infiltrated Tregs numbers and Tregs/CD8+ ratio are associated with adverse prognosis in resectable GC (Shen et al., 2010). On the other hand, a high ratio of CD8+/Tregs in gastric tumors is associated with improved OS of the patients (Liu et al., 2015). Therefore, we determined the association of ROR1 with different types of TIICs in the tumor from GC patients using the EMTome database. As shown in the heatmap, ROR1 showed a strong negative correlation with activated CD8+ and Th17 cells and a positive correlation with plasmacytoid dendritic cells (pDC), Th2, and mast cells in several malignancies (Supplementary Figure S4). In addition, ROR1 showed a moderate negative correlation with activated CD4+, neutrophils, and natural killer T (NKT) cells in a few cancer types (Supplementary Figure S4). In STAD, a moderate to strong negative correlation was obtained between ROR1 and activated CD4+ (rho = -0.497), activated CD8+ T (rho = -0.524), Th17 (rho = -0.28), and NKT cells (rho = -0.169) (Figure 5A-D), whereas a positive correlation of ROR1 was obtained with Treg and macrophages (φ).

M2φ has been shown to promote the progression of GC with peritoneal dissemination and associate with poor prognosis of the patients (Yamaguchi et al., 2016; Liu et al., 2019). Therefore, we explored the association of ROR1 with M2φ and Tregs in GC patients using the EMTome database. As shown in Figure 5E-F, a strong positive correlation was obtained between ROR1 and Tregs infiltration (rho = 0.429) and ROR1 and φ infiltration (rho = 0.394). To determine the type of φ associated with ROR1 expression, we performed the correlation analysis by TIMER 2.0, employing multiple algorithms. As shown in Figure 5G-H, ROR1 expression was positively correlated with M2φ.

*ROR1 and ROR2 genes harbor several missense mutations*

**Figure 6:**
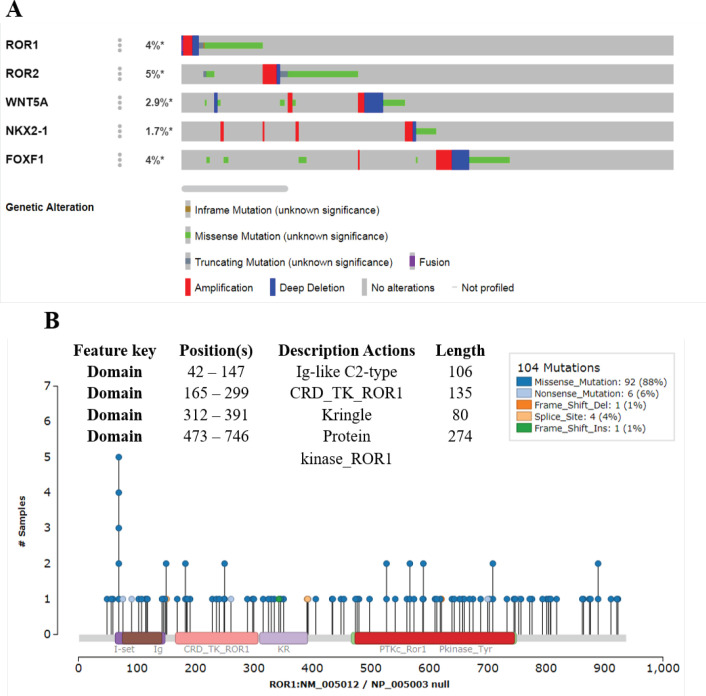
Genetic Alteration in Genes Coding for *ROR1, ROR2, WNT5A, NKX2-1* and *FOXF1* were Analyzed in GC Patients (N=1512) by cBioportal Database. A, data is shown in the Oncoprint format. B, Distribution of somatic mutations in the domains of ROR1 was analyzed by EMTome

We analyzed genomic alterations by cBioPortal on publicly available whole-exome sequencing data of GC. Genomic alterations were noted in *ROR1, ROR2, *and *FOXF1 *in more than 4% of samples, whereas WNT5a and NKX2-1 were altered in 2.9% and 1.7%, respectively, in GC patients (Figure 6). In the case of ROR1, ROR2, and FOXF1, mainly these alterations were missense mutations (Figure 6A). Next, to determine the domains of ROR1 protein affected by the genomic alterations, we performed somatic mutation analysis of the ROR1 gene in GC patients by the EMTome database. As shown in Figure 6B, a total of 104 mutations have been found, and most of the mutations (88%) in the ROR1 are of missense type. Several mutations are present in the kinase domain of ROR1 (473aa to 746 aa). These results suggest that missense mutation could activate the ROR1 constitutively.

**Table 1:** Correlation between mRNA Expression of *ROR1/2, NKX2-1, FOXF1*, and *WNT5A* Genes and Different forms of Survival Outcome in GC Patients

Gene	Survival	Patients	Hazard ratio	95% CI
*ROR1*	OS	875	1.41	1.91-1.68
	FP	640	1.43	1.17-1.76
	PPS	498	2.15	1.71-2.7
*ROR2*	OS	875	1.41	1.23-1.73
	FP	640	1.42	1.16-1.74
	PPS	498	1.75	1.36-2.12
*WNT5A*	OS	875	1.04	0.88-1.23
	FP	640	1.24	0.01-1.52
	PPS	498	0.98	0.78-1.22
*NKX2-1*	OS	875	1.44	1.21-1.71
	FP	640	1.37	1.12-1.68
	PPS	498	1.55	1.24-1.93
*FOXF1*	OS	875	1.42	1.2-1.68
	FP	640	1.33	1.08-1.63
	PPS	498	1.5	1.25-1.93

Footnote: Overall survival (OS), first progression (FP) and post-progression survival (PPS); ROR1/2, receptor tyrosine kinase-like orphan receptor 1/2; NKX 2-1, NK2 Homeobox 1; FOXF1, Forkhead box F1; Wnt5a, Wnt family member 5A

## Discussion

There is a lot of heterogeneity in GC, and it exhibits phenotypic diversity in terms of aggressiveness, response to adjuvant therapy, and survival outcome. The diffuse-type of GC is poorly differentiated, infiltrated with stromal cells, aggressive than the intestinal type, and is associated with a poor prognosis (Ling et al., 2020). Each subtype further shows heterogeneity at the molecular level, and studies are underway to identify more relevant genomic subsets (Lordick and Janjigian, 2016). In the current study, we show that high expression of ROR signaling genes, including *ROR1, ROR2, NKX2-1,* and *FOXF1*, is associated with a worse GC prognosis. The patient cohort with high expression of these genes exhibits shorter OS than the patients with low expression of these genes. A distinct association of ROR1 was noted in the main histological types of GC, intestinal and diffuse types. A highly significant association of ROR1 expression was noted with the OS of GC patients with intestinal-type, but not the diffuse type.

Wnt signaling has emerged as one of the main pathways implicated in cancer development and progression (Menck et al., 2021). The components of the Wnt5a-ROR signaling pathway have been shown to overexpress in several hematological and non-hematological malignancies, including breast, colon, melanoma, lung, and pancreatic cancer (Diamanti et al., 2019; Enayati et al., 2019; Menck et al., 2021). Consistent with our in silico data, previous studies have also demonstrated the prognostic role of Wnt5a-ROR signaling genes with several types of cancer. For example, chronic lymphocytic leukemia patients with high ROR1 expression had significantly shorter therapy-free survival and OS, suggesting that ROR1 is associated with more aggressive disease (Cui et al., 2016). Similarly, ROR2 expression correlates with tumor stage and metastasis in lung, cervical, and breast cancer (Menck et al., 2021) and is associated with worse OS in several types of cancer (Saleh et al., 2019).

Mechanistically, it has been shown that mesenchymal stem cell (MSC)-derived CXCL16 promotes the progression of GC cells by inducing the expression of ROR1through the STAT3-mediated pathway (Ikeda et al., 2020). Interestingly, ROR1 has been identified as a bona fide target of miR-27b-3p, which was downregulated in GC patients, resulting in the upregulation of ROR1 (Tao et al., 2015). miR-27b-3p inhibits GC cell proliferation, colony formation in soft agar, and xenograft tumor formation (Tao et al., 2015). Furthermore, ROR1 has been identified as a transcriptional target of homeobox protein NKX-2, which is required for sustained EGFR signaling in lung cancer patients and serves as an independent prognostic predictor of OS (Yamaguchi et al., 2012; Menck et al., 2021). Indeed, in our study, NKX2-1 showed a high prognostic value in GC patients, correlating with ROR1 expression. Wnt5a is a transcriptional target of FOXF1, a family of transcription factors that regulate embryonic development and is implicated in carcinogenesis. Single nucleotide polymorphisms in FOXF1 have been shown to be associated with GC (Matsusaka et al., 2018). Activation of Wnt5a-ROR2 signaling in MSCs results in enhanced secretion of CXCL16 from MSCs that act on CXCR6 expressed on GC cells and promoted their proliferation (Takiguchi et al., 2016).

TIICs are indispensable components of the TME and play important roles in tumorigenesis, progression, invasion, metastasis, and response to immunotherapy (Zhang et al., 2020). In our study, a negative correlation of ROR1 with activated CD4+, CD8+, and NKT cells observed in GC patients. These findings suggest that the high expression of ROR1 might inhibit the infiltration of these cells into the tumor core, resulting in an aggressive phenotype. In our study, ROR1 expression positively correlated with Tregs and negatively correlated with Th17 cells. Consistent with our findings, abundant infiltration by Tregs and a high proportion of intratumoral Tregs/CD4 ratio (Lee et al., 2018) or Tregs/CD8 ratio (Shen et al., 2010) are associated with poor prognosis of patients with GC.

Tumor-associated macrophages (TAMs) are the most prominent immune cells in TME. Based on the molecular markers and effector function, TAMs are classified into two categories, i) classically activated φ (M1φ) and ii) alternatively activated φ (M2φ). M1φ secrete molecules that prevent the proliferation of surrounding cells, and M2φs release cytokines that favor the proliferation of cancer cells (Ngabire et al., 2020). Notably, a positive correlation between ROR1 with M2φ was noted in our study. Furthermore, M2φ shave been shown to promote cell proliferation, metastasis, angiogenesis, and immunosuppression (Hao et al., 2012). Wnt5A secreted from tumor cells promotes the differentiation of naïve Mφ into M1φ, whereas it inhibits the differentiation into M2 type and T cell activation. Wnt5a also stimulates the secretion of IL10 by DC and Mφs, and IL-10 promotes the differentiation of naïve CD4+ T cells into Tregs (Lopez-Bergami and Barbero, 2020). Significantly, owing to the higher expression of ROR1 in NSCLC and triple-negative breast cancer ROR1 has been employed as a target of chimeric antigen receptor (CAR) T cell therapy (Srivastava et al., 2021). Notably, ROR1-targeting CAR T cells undergo robust expansion in the circulation but fail to accumulate or persist in ROR1+ tumors, and that this failure correlates with the upregulation of multiple coinhibitory receptors and loss of reactivity to ex vivo stimulation (Srivastava et al., 2021).

In conclusion, we show that Wnt5a-ROR signaling genes, especially ROR1, ROR2, NKX2-1, and FOXF1, could serve as a prognostic marker for the intestinal type of GC. Whether the expressions of these genes determine immunotherapy response in GC patients needs to be explored.

## Author Contribution Statement

AK: Conception, study design, interpretation of data, and critical reading and intellectual assessment of manuscript. RN: Analysis and interpretation of data, preparation of the manuscript
